# Case report: Eosinophilia as a rare symptom and primary sign of acute lymphoblastic leukemia with translocation (5;14)(q31;q32)

**DOI:** 10.3389/fonc.2026.1840991

**Published:** 2026-07-15

**Authors:** Pearl van Heteren, Franziska Westendorf, Emin Abdullayev, Juliane Menk, Josephine Faltin, Katayoon Shirneshan, Annette Schaefer, Philippe Schafhausen, Georg Lenz, Matthias Stelljes, Walter Fiedler, Bertram Glaß, Snjezana Janjetovic

**Affiliations:** 1 Department of Hematology and Cell Therapy, HELIOS Klinikum Berlin-Buch, Berlin, Germany; 2 Department of Oncology, Hematology and Bone Marrow Transplantation with Section Pneumology, University Cancer Center Hamburg, University Medical Center Hamburg Eppendorf, Hamburg, Germany; 3 Department of Hematology and Medical Oncology, INDIGHO laboratory, University Medical Center Göttingen (UMG), Göttingen, Germany; 4 Department of Medicine A, University Hospital Münster, Münster, Germany

**Keywords:** acute lymphoblastic leukemia, allogenic stem cell transplantation (ASCT), eosinophilia, IL-3, translocation t(5;14)

## Abstract

**Background:**

Acute B-lymphoblastic leukemia (B-ALL) with t(5;14)(q31;q32) is a very rare subtype of B-ALL, accounting for <1% of cases and often presenting with significant eosinophilia but without peripheral blasts. This condition is driven by a t(5;14)(q31;q32) that juxtaposes the immunoglobulin heavy-chain (IGH) enhancer on chromosome 14q32 with the interleukin-3 (IL-3) gene on 5q31. This results in increased IL-3 production and a characteristic eosinophilia. While this translocation is primarily seen in pediatric patients, it rarely occurs in adults.

**Case report:**

We report two cases of a young adult aged 20-25 years and a 30-year-old man with 25 diagnosis of common B-ALL/B-lymphoblastic lymphoma. In one case, the only clinical symptom was back pain, but laboratory results showed leukocytosis with prominent neutrophilia and eosinophilia. The other case presented with fever, chills, diarrhea, vomiting, and myalgia, accompanied by leukocytosis and eosinophilia. No peripheral blasts were detected. FISH analysis revealed rearrangement of the IGH locus (14q32) in both patients, and karyotyping confirmed a t(5;14)(q31;q32). Both patients were treated according to the GMALL (German Multicenter Study Group for Adult Acute Lymphoblastic Leukemia) recommendations, including allogeneic stem cell transplantation (ASCT). ASCT was performed due to high-risk disease in one case and an early relapse during first-line treatment in the other. During 3 years of follow-up, the first patient remained relapse-free and in good general condition; the second patient died due to septic shock on day 16 after ASCT.

**Conclusion:**

B-ALL with t(5;14)(q31;q32) is an extremely rare entity in adults and may present with pronounced eosinophilia in the absence of peripheral blasts, posing a significant diagnostic challenge. As eosinophilia is often interpreted as reactive, this leukemia subtype may be overlooked or diagnosed late. Therefore, unexplained eosinophilia should prompt early bone marrow investigation with flow cytometry and cytogenetic analysis, even when peripheral blood findings do not suggest acute leukemia.

## 1 Introduction

To date, only a few reports have described the clinical presentation of B-cell acute lymphoblastic leukemia (B-ALL) harboring the t(5;14)(q31;q32). This subgroup, which accounts for less than 1% of acute lymphoblastic leukemias, is included in the current fifth WHO classification (1). A comprehensive analysis (2) showed that all patients (8/8) in this distinct subgroup had eosinophilia, but only 25% (2/8) had peripheral blasts at initial diagnosis. Cases of this rare B-ALL with t(5;14)(q31;q32) have been mostly reported in children and young adults. The t(5;14)(q31;q32) juxtaposes the immunoglobulin heavy-chain (IGH) enhancer located on 14q32 with the interleukin-3 (IL-3) gene on 5q31, which results in increased IL-3 production and subsequent eosinophilia (3, 4). Due to eosinophilia, patients can experience symptoms such as thromboembolic events, skin manifestations (e.g., pruritus or erythematous rash), diarrhea, and hepato- and/or splenomegaly. Eosinophilia occurs as a secondary effect of the translocation and is not directly caused by the acute leukemia (5).

Here, we report two cases of young patients diagnosed with B-ALL with t(5;14)(q31;q32) who presented with eosinophilia as the sole peripheral blood abnormality.

## 2 Case description

### 2.1 Case report 1

**Figure 1:**
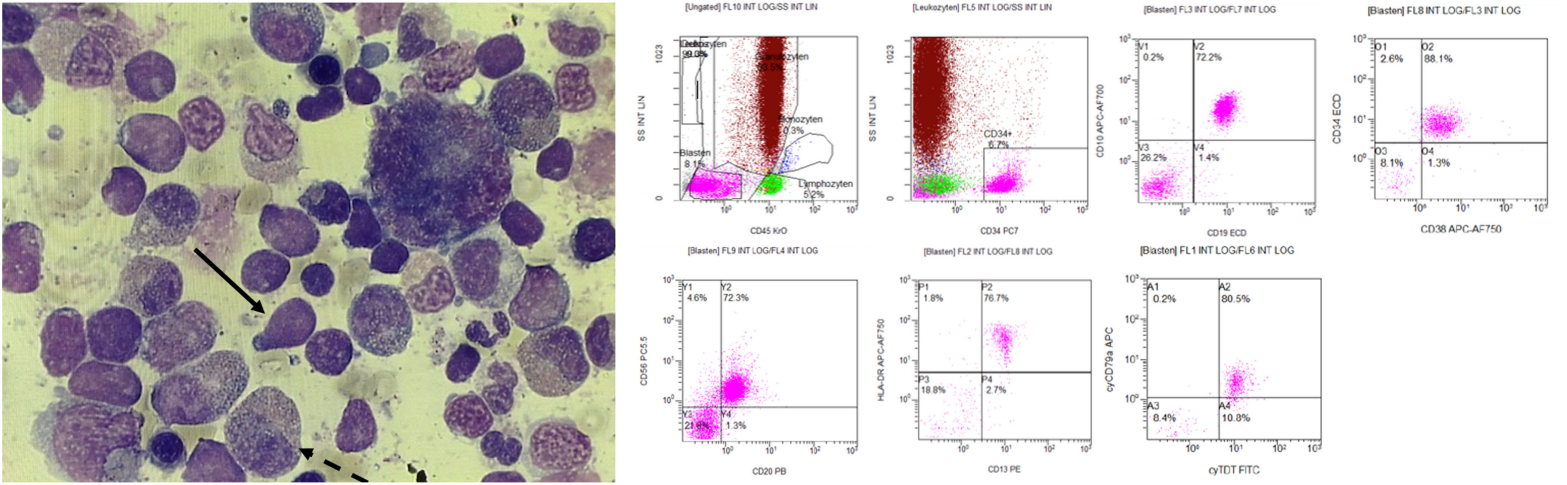
**(a)** Cytomorphology of the bone marrow aspirate from patient 1. Small blasts with scant cytoplasm (solid arrow) and eosinophils (dashed arrow) (May–Grünwald–Giemsa stain). **(b)** Flow cytometry of patient 1, bone marrow. The blasts were positive for progenitor markers CD34, HLA-DR, and cyTDT, and additionally for the B-cell markers CD19, CD20, and CD10, with an aberrant coexpression of CD56, CD38, and CD13.

**Figure 2:**
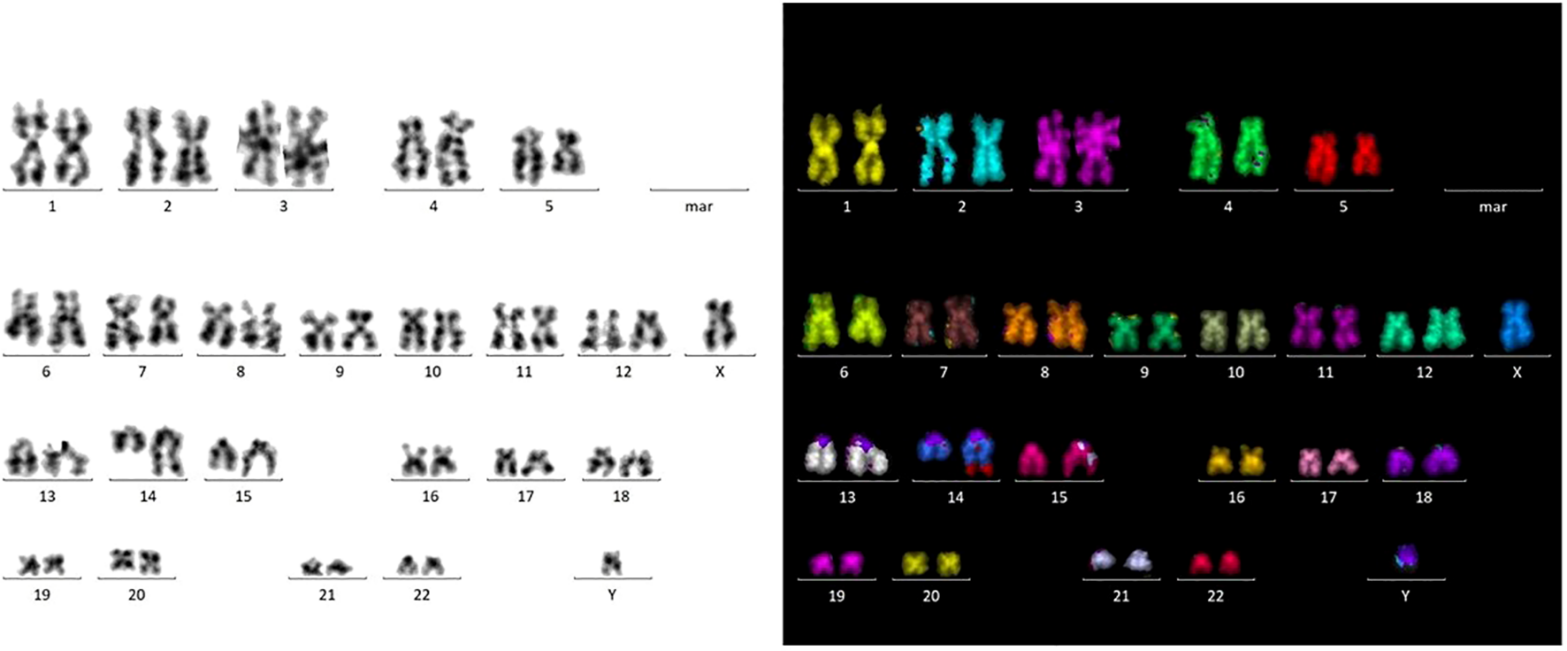
**(a, b)** Conventional cytogenetic analysis (G-banding) and multicolor fluorescence *in situ* hybridization (mFISH) of bone marrow cells from patient 1 at diagnosis, demonstrating a t(5;14)(q31;q32).

A 30-year-old man was admitted to the Department of Hematology and Cell Therapy, HELIOS Klinikum Berlin-Buch, Germany, for further evaluation of unexplained leukocytosis with hypereosinophilia and neutrophilia. He reported 3 months of back pain on standing and walking, and had a pre-existing condition of bronchial asthma. Computed tomography (CT) showed axillary, retroperitoneal, inguinal, and parailiac lymphadenopathy with concomitant splenomegaly (14.3 cm). Additionally, a pathological fracture of the second lumbar vertebra with osteolysis was observed. Routine blood sampling showed leukocytosis of 69.05 Gpt/L (billion cells per liter; normal range: 3.9–10.2 Gpt/L), predominantly neutrophilia at 54.2 Gpt/L (normal range: 1.5–7.7 Gpt/L), and eosinophilia at 7.54 Gpt/L (normal range: 0.02–0.5 Gpt/L). No left shift or blasts were detected in the peripheral blood. Hemoglobin level was 14.5 g/dL (grams per deciliter; normal range: 13.5–17.2 g/dL) and platelets were 236 Gpt/L (normal range: 150–370 Gpt/L). Physical examination and diagnostic investigations showed no evidence of eosinophilia-induced tissue injury. Common causes of eosinophilia, such as atopic diseases, parasitic infestations, or autoimmune diseases, were ruled out. Bone marrow aspiration revealed hypercellular bone marrow with 50% blasts and 30% dysplastic eosinophilic granulocytes (cytologically, **Figure 1a**). Granulopoiesis was decreased but showed maturation to segmented nucleated granulocytes. Flow cytometry showed blastic cells expressing progenitor markers (CD34, CD117, and TdT), as well as B-lymphoid antigens (CD10, CD19, and CD20). Additionally, aberrant coexpression of myeloid markers (CD13, CD15, and CD56) was observed (**Figure 1b**). Histopathology confirmed lymphoid blast proliferation with expression of CD4, TdT, PAX5, CD19, and CD20. Based on these results, acute lymphoblastic leukemia, common B-ALL subtype, was diagnosed. Strikingly, an increase in mast cells with aberrant CD25 expression was noted in the bone marrow. However, neither increased tryptase nor a c-Kit-D816V mutation was detected, thereby ruling out systemic mastocytosis with associated hematologic disease. Fluorescence *in situ* hybridization (FISH) demonstrated involvement of the IGH locus in a chromosomal rearrangement. Conventional karyotyping subsequently confirmed a translocation (5;14)(q31;q32) (**Figures 2a, b**). Other molecular screening analyses, including next-generation sequencing (NGS), showed no pathological results.

Three days after initial presentation, the patient was treated according to the GMALL consensus protocol (6) with a prephase of dexamethasone (10 mg/m^2^, d1-5) and cyclophosphamide (200 mg/m^2^, d3-5). This treatment led to a rapid decrease in white blood cells. In the subsequent induction therapy, the patient received rituximab (375 mg/m^2^, d5), dexamethasone (10 mg/m^2^, d6 + 7, d13-16), vincristine (2 mg abs., d6, 13, 20), daunorubicin (22.5 mg/m^2^, d6 + 7, d13 + 14), and PEG-asparaginase (2,000 IE/m^2^, d20). Due to an allergic reaction with hypotension, PEG-asparaginase was replaced by Erwinase (20,000 IE/m^2^, d23 + 30 + 33). The frequency of administration was reduced due to elevated liver enzymes. After this induction therapy, the patient achieved complete remission by day 22, with undetectable t(5;14)(q31;q32) and no cytological or flow cytometric signs of blasts. Without complications, the patient then received induction 2, consisting of rituximab (275 mg/m^2^, d23 + 44), cyclophosphamide (1 g/m^2^, d24 + 44), cytarabine (75 mg/m^2^, d26-29, 33-36, 40-43), and Erwinase (20,000 IE/m^2^, every other day, d35-43). To prevent central nervous system (CNS) involvement, he received intrathecal triple therapy (MTX 15 mg abs., cytarabine 40 mg abs., dexamethasone 4 mg, 5 times) and CNS radiation. After the second induction (week 9), an ongoing complete molecular [PCR-based analysis of T-cell receptor gamma (TRG) gene rearrangements] and cytological remission of B-ALL was observed. Due to the high-risk ALL (according to GMALL, with hyperleukocytosis >30 Gpt/L at initial diagnosis), an allogeneic stem cell transplantation (ASCT) was performed as consolidation therapy 134 days after treatment initiation. The patient received an allogeneic, non-related, human leukocyte antigen (HLA) 10/10-compatible, ABO blood group major- and minor-incompatible peripheral blood hematopoietic stem cell transplantation. He developed several infectious complications, including hepatosplenic candidiasis, hepatitis virus-associated mucositis, and atypical pneumonia; however, he was able to leave the hospital 45 days post-transplant. He developed mild cutaneous graft-versus-host-disease (GvHD), which was managed with topical therapy. Currently, the patient remains relapse-free for more than 5 years after ASCT and is in good general condition.

### 2.2 Case report 2

A 20-25 years-old male patient was referred from the Infectious Disease Department to a Hematology Department at a University Medical Center in the north of Germany due to unexplained eosinophilia (**Figure 2**). Initially, the patient presented with fever, myalgia, chills, nausea, vomiting, and diarrhea following an overseas trip and received treatment for suspected helminthiasis in the infectious disease department. The symptoms improved, yet eosinophilia persisted. Consequently, a bone marrow biopsy was performed, revealing a CD20-negative B-lymphoblastic lymphoma with 23% blasts in the bone marrow smear. No blasts were observed in the peripheral blood. Abdominal ultrasound showed hepatosplenomegaly without lymphadenopathy, and chest X-ray was unremarkable; no peripheral lymphadenopathy was detected. No other eosinophil-related tissue injury was found. Flow cytometry identified blasts positive for CD19, CD10, CD34, HLA-DR, CD22, cyCD79a, and TdT, while negative for CD20. Cytogenetic analysis showed a t(5;14)(q31;q32) in 4 metaphases out of 11 normal metaphases. No Philadelphia chromosome was detected, and *FIP1L1-PDGFRA*-fusion was ruled out, establishing a diagnosis of CD20-negative B lymphoblastic lymphoma with a distinct karyotype. There was no evidence of CNS involvement. Prephase treatment was initiated immediately after diagnosis confirmation. Definitive treatment, as part of the GMALL 07/2003 study protocol (6), commenced 163 days after initial presentation. Further treatment according to protocol was administered closer to the patient’s home, including CNS prophylaxis with intrathecal chemotherapy and prophylactic cranial irradiation during induction phase II. After induction phase II, cytological remission was achieved. However, following planned consolidation therapy I, an early cytological relapse with 8% blasts in the bone marrow was detected 109 days after the start of ALL treatment. Treatment continued with consolidation II and III according to GMALL 07/2003 protocol, followed by a matched unrelated donor ASCT due to the early relapse 208 days after treatment initiation. The patient died on day 16 after ASCT due to severe septic shock.

## 3 Discussion

ALL with translocation (5;14)(q31;q32) remains a very rare disease that affects less than 1% of all acute lymphoblastic leukemias (7). However, due to its unique presentation and genetic changes, it is described as an independent entity according to WHO classification (1). The fusion of gene locus 5q31, which encodes IL-3, with gene locus 14q32, encoding IGH, results in the activation and overexpression of IL-3. This leads to a proliferation of eosinophils, which are not classified as blasts but rather considered a “by-product” of the gene translocation (5). Given the eosinophilia and its clinical spectrum, including skin, CNS, or cardiac involvement (8, 9), patients often become clinically symptomatic early, even before blasts are detectable in the peripheral blood.

In cases of unclear eosinophilia, differential diagnoses such as reactive eosinophilia due to infections, tissue-invasive parasites, or various other triggers (allergy, autoimmune diseases, atopy, lymphomas, etc.) should be ruled out (10). The diagnostic workup for eosinophilia also includes screening for other malignant bone marrow diseases accompanied by eosinophilia (11, 12).

Peripheral blood eosinophilia, typically presenting with normal hemoglobin and platelet counts and an absence of blasts, is a characteristic finding (2). Therefore, bone marrow aspiration should be considered to clarify eosinophilia of unknown origin, especially in young adults and children.

The mean bone marrow blast count in adult patients with ALL with t(5;14)(q31;q32) was 41.1% (range: 0%– 80%, **Supplementary Table 1**), which explains the largely preserved hematopoiesis with normal platelet and hemoglobin counts at the time of diagnosis (2). In addition to cytomorphology and flow cytometry, cytogenetic and molecular genetic analyses should be performed for initial diagnostics. However, some reports demonstrated that it can be difficult to identify the t(5;14)(q31;q32) even with high blast counts using chromosome banding analysis, because the translocation is often identified in only a few metaphases (13, 14). Therefore, FISH analysis should also be performed. However, it should be stressed that the translocation is present only in blasts, not in eosinophils. If the blast count is low, FISH analysis may yield false-negative results with a normal karyotype. Therefore, modern molecular techniques such as next- generation sequencing (NGS) should be performed additionally (2). NGS can provide deeper coverage and identify cryptic rearrangements that may be missed by FISH, especially when the specific translocation is rare or present in a low proportion of cells. Furthermore, targeted NGS panels that include the IGH locus enable more reliable detection of IGH translocations compared to conventional methods. In some case reports, only an extended diagnosis using NGS for an IL3-IgH rearrangement led to the correct diagnosis (12–14).

**Figure 3:**
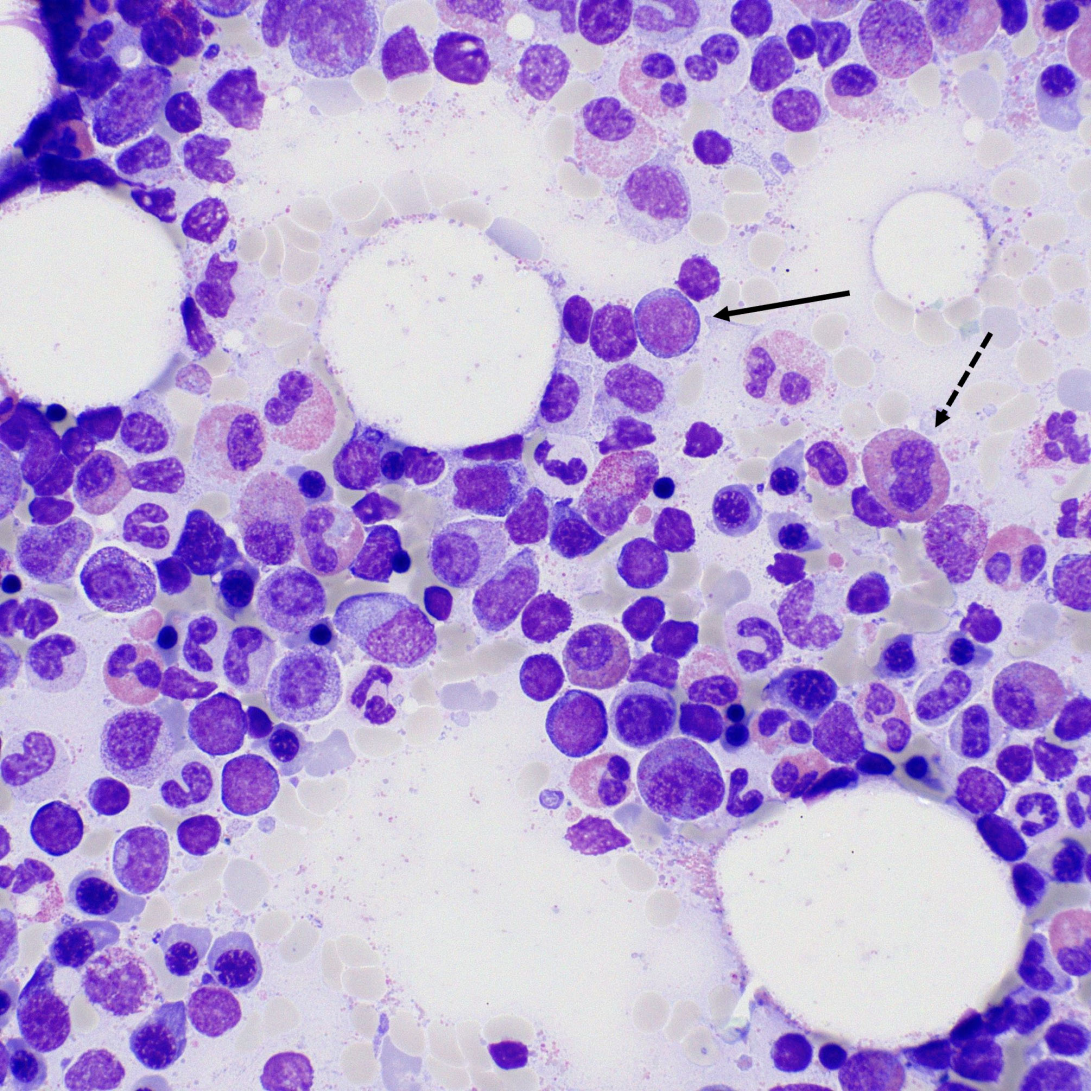
Bone marrow aspirate smear at diagnosis of patient 2 showing eosinophils (dashed arrow) and lymphoblasts in lymphoblastic leukemia (solid arrow) with t(5;14)(q31;q32) (May–Grünwald–Giemsa stain).

**Figure 4:**
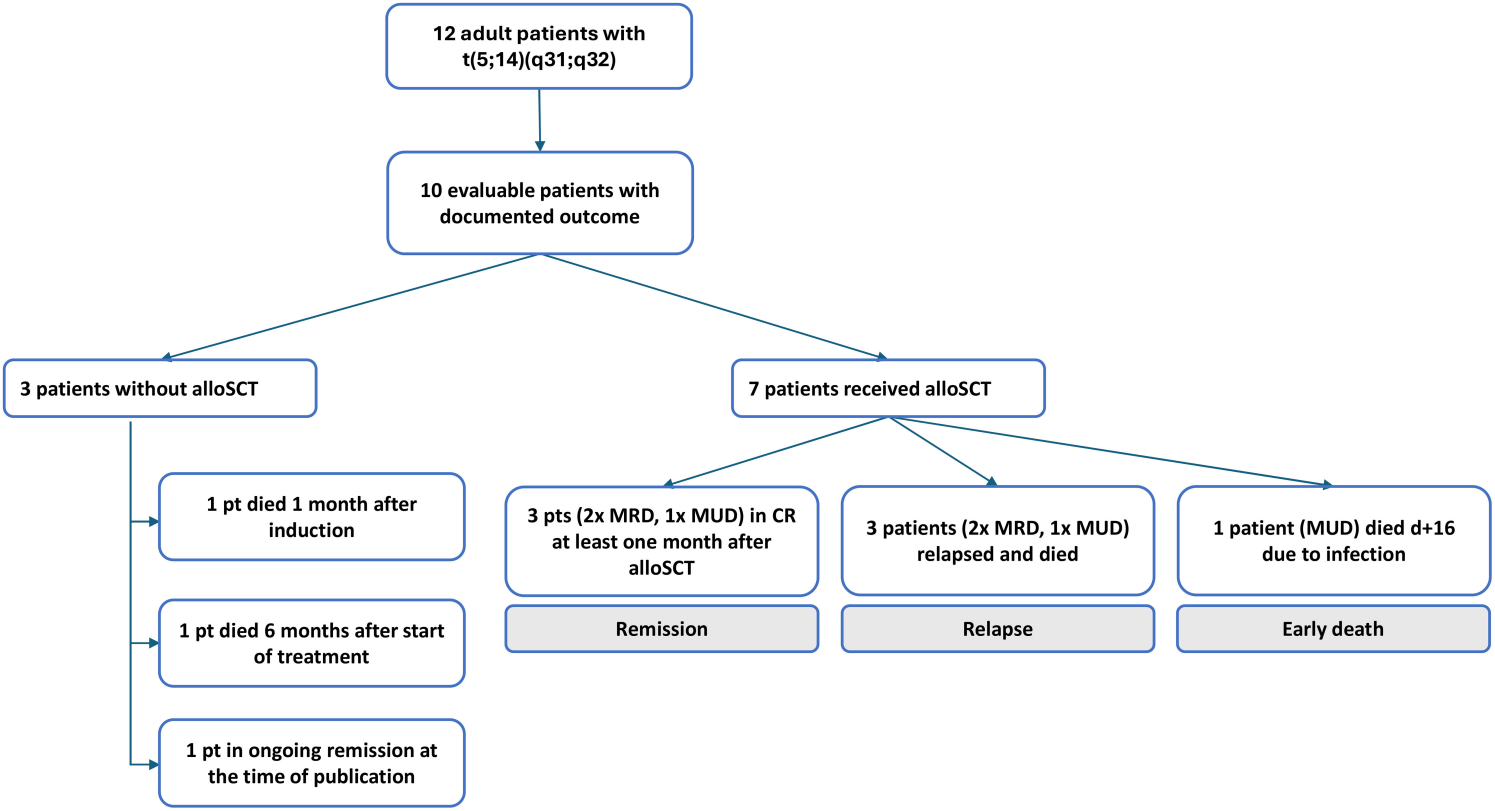
Flowchart of adult patients (pts) with t(5;14)(q31;q32). ASCT, allogeneic stem cell transplantation; CR, complete remission; MRD, matched related donor; and MUD, matched unrelated donor.

To date, only 12 adult patients with acute B-cell lymphoblastic leukemia and t(5;14)(q31;q32) have been reported (**Figure 4**). Patient characteristics, including those of our two patients, are summarized in **Supplementary Table 1**. The median age was 30.5 years (range: 18–60 years), with a male predominance (10:2). At disease onset, all patients presented with leukocytosis (mean 49.9 × 10^6^/L, range: 15.9–114 × 10^6^/L) and marked eosinophilia (median 29.2 × 10^6^/L, range 6–96 × 10^6^/L), which accounted for a mean of 59% of leukocytes. Several patients exhibited symptoms likely related to eosinophilia, such as deep vein thrombosis or arterial embolic events (4/12). Fever (6/12), weight loss (2/12), and back pain (2/12) were also attributed to acute B-cell leukemia. Remarkably, almost no blasts were detectable in the peripheral blood of these patients (median 0, range 0–2), whereas the mean blast count in bone marrow was 42% (range 0–80%). Ten of twelve patients received induction therapy (two cases not documented) for acute lymphoblastic leukemia. One patient died 1 month after the start of induction therapy; two others were treated with chemotherapy alone, one of whom achieved ongoing remission. For consolidation, seven of twelve patients underwent ASCT (four with a matched related donor, two with a matched unrelated donor; data for one patient were not available). Outcomes after –ASCT were documented for five of the seven patients (**Figure 3**). Three patients experienced fatal outcomes: one had an isolated CNS relapse and died 12 months after diagnosis of relapse; another passed away 10 months after transplantation due to relapse and septic shock; and a third patient relapsed 2 years after transplantation. Taken together, three of the six evaluable patients who underwent induction and consolidation therapy experienced relapse between 6 months and 2 years after ASCT. Three patients are still alive, but survival data for two patients are not available. While recognized as a distinct entity in the current WHO classification (1), no specific risk category has yet been defined. Available data from small case series suggest a heterogeneous clinical course. Given the limited available data, its prognostic significance remains uncertain.

## 4 Conclusion

In conclusion, the diagnosis and treatment of B-ALL with t(5;14)(q31;q32) remain challenging. Incorporating bone marrow diagnostics using FISH and NGS is necessary to detect these rare cases in patients with unexplained eosinophilia. Published data for pediatric patients demonstrate ongoing remissions in seven of eight patients (2). In contrast, outcomes in adults remain poor, highlighting the need for improvement and potentially representing a target for the development of novel therapeutic strategies.

## Data Availability

The original contributions presented in the study are included in the article/**Supplementary Material**. Further inquiries can be directed to the corresponding author.

## Supplementary material

Supplementary Table 1 Patient characteristics of adult patients with acute B-cell lymphoblastic leukemia and t(5;14)(q31;q32).
